# Anharmonic DFT Study of Near-Infrared Spectra of Caffeine: Vibrational Analysis of the Second Overtones and Ternary Combinations

**DOI:** 10.3390/molecules26175212

**Published:** 2021-08-27

**Authors:** Justyna Grabska, Krzysztof B. Beć, Yukihiro Ozaki, Christian W. Huck

**Affiliations:** 1 CCB—Center for Chemistry and Biomedicine, Institute of Analytical Chemistry and Radiochemistry, Leopold-Franzens University, Innrain 80/82, 6020 Innsbruck, Austria; Krzysztof.Bec@uibk.ac.at (K.B.B.); Christian.W.Huck@uibk.ac.at (C.W.H.); 2 School of Biological and Environmental Sciences, Kwansei Gakuin University, Sanda 669-1337, Hyogo, Japan; yukiz89016@gmail.com; 3 Toyota Physical and Chemical Research Institute, Yokomichi, Nagakute 480-1192, Aichi, Japan

**Keywords:** near-infrared (NIR) spectroscopy, overtone, combination band, caffeine, anharmonicity

## Abstract

Anharmonic quantum chemical calculations were employed to simulate and interpret a near-infrared (NIR) spectrum of caffeine. First and second overtones, as well as binary and ternary combination bands, were obtained, accurately reproducing the lineshape of the experimental spectrum in the region of 10,000–4000 cm^−1^ (1000–2500 nm). The calculations enabled performing a detailed analysis of NIR spectra of caffeine, including weak bands due to the second overtones and ternary combinations. A highly convoluted nature of NIR spectrum of caffeine was unveiled, with numerous overlapping bands found beneath the observed spectral lineshape. To properly reflect that intrinsic complexity, the band assignments were provided in the form of heat maps presenting the contributions to the NIR spectrum from various kinds of vibrational transitions. These contributions were also quantitatively assessed in terms of the integral intensities. It was found that the combination bands provide the decisively dominant contributions to the NIR spectrum of caffeine. The first overtones gain significant importance between 6500–5500 cm^−1^, while the second overtones are meaningful in the higher wavenumber regions, particularly in the 10,000–7000 cm^−1^ region. The obtained detailed band assignments enabled deep interpretation of the absorption regions of caffeine identified in the literature as meaningful for analytical applications of NIR spectroscopy focused on quantitative analysis of caffeine content in drugs and natural products.

## 1. Introduction

Near-infrared (NIR) spectroscopy is a potent and valued physicochemical technique of analysis [1,2,3]. This technique is primarily recognized in the applications to analytical chemistry [4] as a rapid, cost-efficient, simple, and non-destructive technique with nearly no sample preparation required, which appears to be a very attractive and useful alternative tool for qualitative and quantitative analyses in the agriculture [5,6], food [7,8], chemical [9,10], and pharmaceutical industries [11]. However, it is also met with an increasing importance in bioscience [12,13], medicine [14,15], and various applications related to hyperspectral imaging as well [16]. This method appears also useful in physical chemistry by delivering unique information on the molecular structure, hydrogen bonding, interactions, and dynamics [17,18,19].

The intrinsic complexity of NIR spectra, resulting from a large number of overlapping bands that create a strongly convoluted lineshape [2], makes them difficult for detailed interpretation, which remains a hindrance in a number of applications [20]. Extensive studies established characteristic vibrational frequency ranges and comprehensive band assignment tables for MIR or Raman spectra [21,22]. In contrast, the analogous information available for NIR spectroscopy remains fairly shallow [23]. Because of the anharmonicity, it is difficult to yield an exhaustive interpretation of NIR spectra by means of classical methods [24]. In recent years, significant progress in theoretical methods of calculation of anharmonic spectra was observed [25,26,27]. It was demonstrated that very accurate reproduction of NIR spectra of small- to medium-sized molecules in liquid phase and in solutions is possible [28,29,30], including very fine spectral details resulting from the conformational populations [31,32,33]. NIR spectra simulations were also performed for biologically-relevant molecules, e.g., short- [34,35], medium- [36], and long-chain fatty acids [37], amino acids [38,39] or nucleobases [40]. The theoretical calculations of the spectra were used to directly aid analytical applications of NIR spectroscopy, where the interpretation of the multivariate regression models and understanding of the instrumental difference between various benchtop and miniaturized NIR spectrometers has been made available this way. Relevant examples involved phytopharmaceutical compounds such rosmarinic acid [41], thymol [42], as well as a number of others compounds in scope of a given analytical application [43,44,45]. Furthermore, focused studies on the anharmonicity present in MIR and NIR spectra manifested in the band shifts, intensity variations, and the contributions of the specific types of vibrational transitions to the total intensity in the respective spectral regions [46,47].

The primary focus in the theoretical studies of NIR spectra was directed at the first overtones and binary combinations, as these bands are the most meaningful for shaping NIR spectra and are easier to calculate as well. Recent investigation of NIR and MIR spectra of melamine demonstrated that the simulation of NIR bands corresponding to three quanta excitations, i.e., second overtones and ternary combinations, offer an improved interpretation of the spectra [48]. However, limited attention has been given so far to the short-wavelength fragment of NIR spectra, i.e., above 7000 cm^−1^, which is solely populated by these higher order bands. That region encapsulates rich information on the sample and thus is often highly useful in practical applications, even more so given the popularity of Vis/SW-NIR sensors [49].

In this work we took on the aim to analyze in detail the NIR spectrum of caffeine (1,3,7-trimethylpurine-2,6-dione, C_8_H_10_N_4_O_2_), including the first and second overtones, as well as binary and ternary combination bands in a broad spectral region between 10,000–4000 cm^−1^. Caffeine serves a useful and interesting subject for such study as it reconciles interesting molecular structure for a fundamental study with practical importance in applications [50,51]. With respect to the first aspect, the presence of a purine ring and C = O, C-H, and N-H functional groups in the molecule of caffeine leads to the appearance of highly characteristic NIR absorption peaks. Next, other than coffee [52,53], caffeine occurs naturally in many kinds of plants, and is an important ingredient in many foodstuff and beverages, e.g., in tea [54,55], mate [56,57], cocoa [58], and beverages [59]. Caffeine is an important constituent of many drugs as well, given its broad scope of therapeutic properties [60,61,62]. Following its broad significance for agro-food and pharmaceutical sectors, rich literature on the chemical analysis of the samples containing caffeine analysis is available, e.g., using UV-VIS [63,64], HPLC [65], or NMR [66] modalities. Importantly, among the vibrational spectroscopic methods including MIR [67] and Raman [68,69] techniques, NIR spectroscopy is a particularly potent tool for the assessment of coffee quality characteristics [70,71,72,73]. In the analytical NIR spectroscopic studies, the characteristic absorption regions of caffeine have been discussed in relation to their accessibility resulting from overlapping with the regions where the signals from other constituents appear [51,74,75]. However, only limited anticipatory information on the physical origins of these absorption regions have been available so far [51]. One of the aims of the present investigation is to provide practical impact as well, by providing the interpretation of the wavenumber regions that have been found in the literature to be the most meaningful for the multivariate calibration models constructed for analysis of caffeine in various cases.

Finally, the second overtones and ternary combination bands provide a very useful source of structural information in physicochemical studies. The second overtones deliver important information on the anharmonicity constant, supplementing the one available from the analysis of the first overtones, yielding useful insights into intermolecular interactions and solvent effect [76,77]. The second overtone peaks are better separated and also appear clearly in the upper NIR region, free from the strong absorption from other bands. Further, the ternary combinations are particularly numerous types of transitions expressed in the spectra, as the number of the possible co-excitations is far greater than in the lower order combinations [78]. However, for this reason and the fact that these are very weak bands, their analysis is extremely difficult and, to our best knowledge, only few of our previous studies attempted to extract structural information from ternary combinations [33,48,79,80,81].

## 2. Results and Discussion

### 2.1. Analysis of a NIR Spectrum of Caffeine

**Figure 1:**
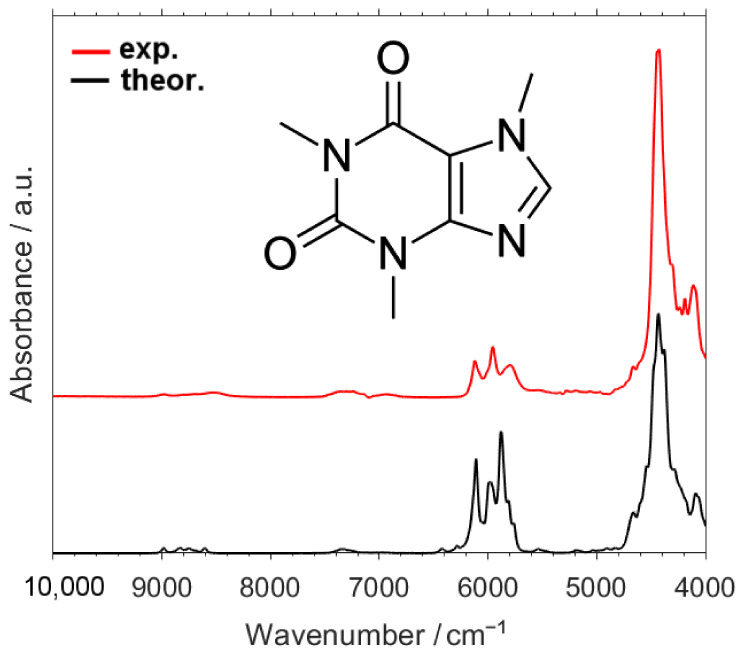
Experimental and calculated NIR spectra of caffeine in the region of 10,000–4000 cm^−1^.

**Figure 2:**
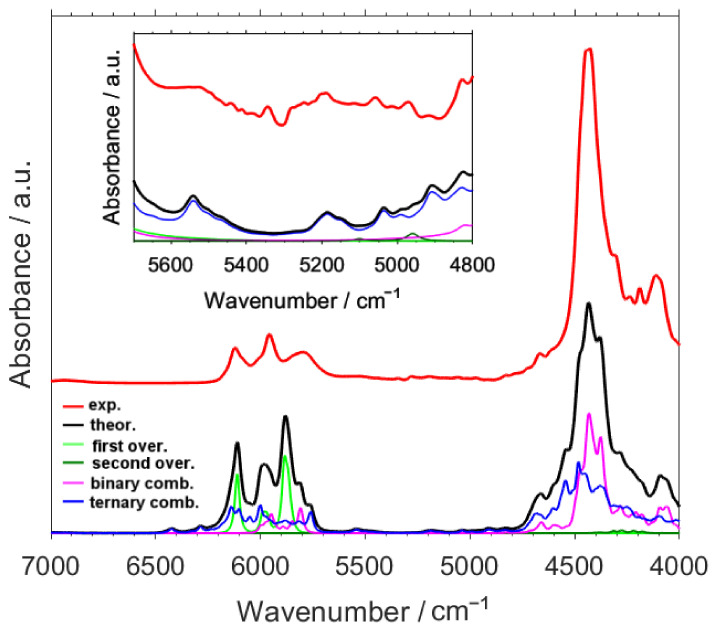
Experimental and calculated NIR spectra of caffeine in the region of 7000–4000 cm^−1^. Theoretical lineshapes representing the summed bands of different origin are presented. Refer to Figures S2–S5 in Supplementary Material for better view of details.

**Figure 3:**
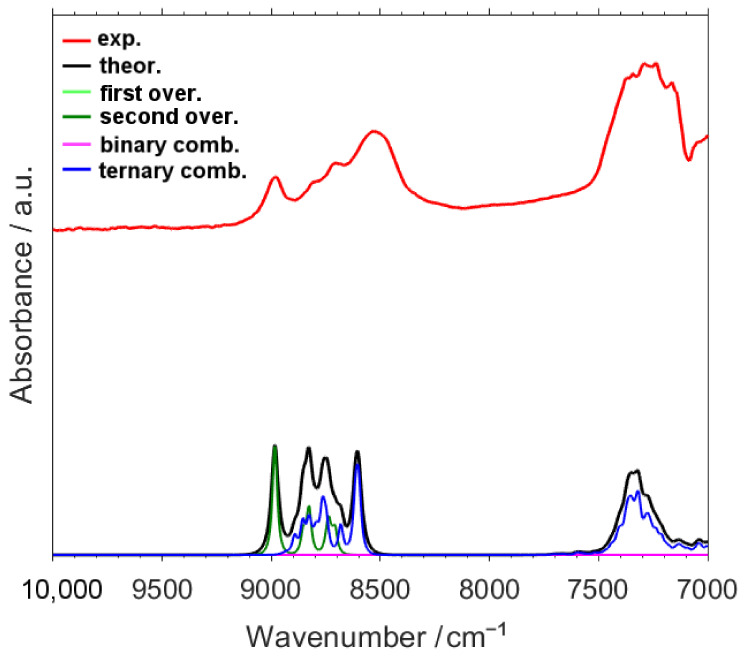
Experimental and calculated NIR spectra of caffeine in the region of 10,000–7000 cm^−1^. Theoretical lineshapes representing the summed bands of different origin are presented. Refer to Figures S6–S8 in Supplementary Material for a better view of details.

As depicted in Figure 1, the calculated NIR spectrum of caffeine reproduces the experimental one very well, including fine absorption features visible near the shoulders of the major bands in 7000–4000 cm^−1^ region (Figure 2), as well as the weak peaks observed in 10,000–7000 cm^−1^ region (Figure 3). Thus, a detailed and reliable interpretation of the spectrum of caffeine is possible. An extensive level of band overlap is observed in the NIR spectrum, as presented in detail in Figure S1 in Supplementary Material. In Figure 2 and Figure 3 (and in Figures S2–S8), in addition to the theoretical lineshape, the contributing lineshapes corresponding to the different vibrational transitions, i.e., first and second overtones as well as binary and ternary combinations, are presented. This opens the opportunity to dissect the NIR lineshape with respect to the underlying bands and to elucidate their relationships with the structural features of caffeine.

The presence of the first overtone bands is primarily noticeable in the peaks appearing at 6200–5800 cm^−1^, albeit the contributions from both binary and ternary combinations are clearly visible there as well (Figure 2, Figure S5). The most evident manifestation of the second overtones is seen in the bands visible at ca. 9100–8500 cm^−1^ (Figure 3 and Figure S7). Throughout the entire spectrum, the contributions from combination bands is well recognized, e.g., within the main absorption features observed between ca. 4800–4000 cm^−1^ and 6100–5800 cm^−1^ (binary and ternary combinations; Figure 2, Figures S2 and S5) or ca. 7400–7000 cm^−1^ and 9100–8500 cm^−1^ (ternary combinations; Figure 3 and Figures S6–S8).

**Figure 4:**
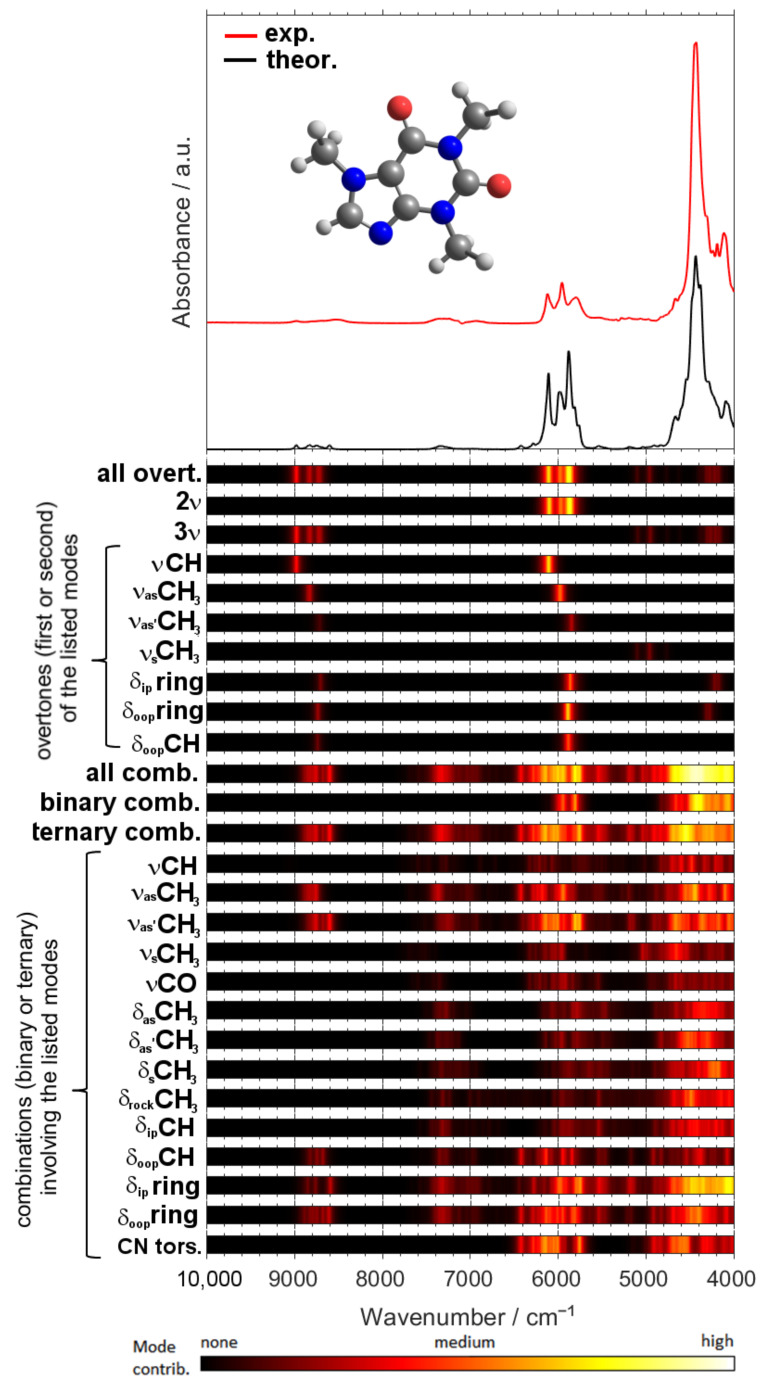
Analysis of the contributions to NIR spectrum of caffeine based on the calculated spectrum (GVPT2//B3LYP-GD3BJ/SNSD).

The relative significance of the different types of vibrational transitions in NIR spectrum of caffeine, with respect to a given wavenumber region of interest, may be quantitatively assessed by calculating the integral intensity for the simulated lineshapes (as presented in Figure 3 and Figure 4). These contributions are collected in Table 1. One should easily notice that in the NIR spectrum of caffeine, the overtones of either kind are significantly less meaningful than the combination bands (Table 1). The first overtones contribute only by 11.57% to the entire analyzed spectral region; furthermore, these bands are mostly limited to the 6500–5500 cm^−1^ region, as their contribution outside this narrow fragment of the spectrum is negligible. However, within that narrow wavenumber range (6500–5500 cm^−1^) the contribution of the first overtones is clearly articulated (40.5%).

In the case of second overtones, their presence in the spectrum is merely noticeable with slightly more than just 1% of the total contribution. Those transitions are only meaningful for the absorption in the region above 7000 cm^−1^ (26.78%). However, in the higher wavenumber region of NIR spectrum, between 9200–8400 cm^−1^, the second overtones are highly meaningful, with the calculated 42.3% of the contribution to the total integral intensity in that region. Noteworthy, the overtone bands of caffeine present a different picture to that of O-H or N-H group bearing molecules, which were very often examined by NIR spectroscopy [17,19,26,31,77,81]. The overtones of O-H and N-H stretching vibration often appear as strong, well-resolved peaks observed in narrow wavenumber regions. These features enabled them to be used as a useful source of information on the structure and interactions, especially hydrogen-bonding [17,19,82]. However, in the caffeine case, the first overtones overlap with combination bands (6500–5500 cm^−1^; Figure 2), and the second overtones mostly do as well (ca. 8900–8600 cm^−1^; Figure 3), with the exception of a relatively sharp second overtone of C-H stretching observed at 8980 cm^−1^ (Figure 3; Table 2). Therefore, in the cases such as the caffeine examined here it is necessary to perform a full simulation of NIR spectra, including both overtones and combinations, to successfully extract the structural information.

An interesting observation can be made in the case of the combination bands of caffeine. In contrast to the earlier studied compounds where the binary combinations were relatively more significant [79,83], in the present case the greatest contribution to the total intensity stems from the ternary combinations, with 50.24% calculated for the 10,000–4000 cm^−1^ region. This results from a significant presence of the ternary combinations in ca. the 5000–4000 cm^−1^ region of caffeine, which for the earlier investigated cases was far more decisively influenced by binary combinations [breakthrough]. Noteworthy, the ternary combination bands are solely responsible for the absorption of caffeine in 7700–7000 cm^−1^ region (99.95% of the total integral intensity).

As one should anticipate, the higher wavenumber NIR region of caffeine (10,000–7000 cm^−1^) is entirely populated by the second overtones and ternary combination bands. Interestingly, while the NIR spectrum of caffeine is decisively influenced by the combination modes, the importance of the overtones increases towards the higher wavenumbers, as shown in Table 1. Considering the upper narrow fragment of the NIR region, 9200–8400 cm^−1^, the contributions stemming from these two types of transitions tends to equalize with 42% and 58% attributed to the overtones and combinations, respectively. Hence, including these bands in the spectra simulation is essential to interpret the meaningful variables for the analysis of caffeine content, which have been reported to extend up to 9996 cm^−1^ [50] and 10,000 cm^−1^ [84].

### 2.2. Detailed Interpretation of NIR Bands of Caffeine

As the consequence of the highly convoluted nature of NIR spectra (Figures S1, S3, and S6 in Supplementary Material), the typical approach to present tabularized band assignments, as it is commonly done in MIR spectroscopy, is not feasible. Instead, the assignments given in the form of heat maps, corresponding to the extent of the contribution of a given transition, better represent the origin of NIR absorption (Figure 4). In this way of presentation, the ratio between the intensity of a given simulated individual band to the total intensity of the summarized theoretical lineshape is calculated for each spectral point (i.e., wavenumber). The resulting value is then coded using a colormap for ease-of-view, as presented in Figure 4. This way, the observed lineshape may be better understood, as it originates from numerous overlapping individual bands. However, for clarity, a table that summarizes the assignments for the major NIR peaks of caffeine is additionally provided (Table 2).

The region of the most intense bands, ca. 5000–4000 cm^−1^ features an extremely high extent of overlapping binary and ternary combination bands. Numerous individual modes can be involved in the bands observed there (Figure 4). Interestingly, the very weak second overtone bands contribute by miniscule but noticeable extent here, e.g., the second overtone of in-plane (3δ_ip_ring) and out-of-plane ring deformation mode (3δ_oop_ring) in the 4400–4200 cm^−1^ or 3ν_s_CH_3_ in 5200–4900 cm^−1^ region. Noteworthy, it is evident that the contributions to the NIR spectrum from the low-frequency δ_oop_ring mode becomes possible through the ternary combination transitions, as either the first overtones or binary combinations are located in MIR region.

The origin of the next distinguishable absorption of moderate intensity, between ca. 6200–5800 cm^−1^, can be firstly assigned to binary and ternary combinations, primarily with the ring deformation and methyl stretching modes involved: δ_oop_ring, δ_ip_ring, ν_as_CH_3_, and ν_as_’CH_3_. Secondly, the first overtones of C-H stretching and ring deformation modes, i.e., 2νCH, 2ν_as_CH_3, 2_ν_as’_CH_3, 2_δ_ip_ring, 2δ_oop_ring, and 2δ_oop_CH, contribute in this region. The region of very weak intensity, between ca. 5800–5000 cm^−1^, mostly arises from the ternary combinations mostly constituting δ_ip_ring and δ_oop_ring modes, among others.

In the 7400–6800 cm^−1^ region one can observe ternary combinations with δ_ip_ring, δ_oop_ring and ν_as_CH_3,_ and ν_as’_CH_3_, as well as lesser contributions from other deformation modes of CH_3_ group. The absorption in the upper NIR region, ca. upper 9100–8700 cm^−1^, consists of second overtones (mostly 3νCH, 3ν_as_CH_3_, 3ν_as’_CH_3_, 3δ_ip_ring, 3δ_oop_ring, and 3δ_oop_CH) and ternary combinations (primarily δ_ip_ring, δ_oop_ring, δ_oop_CH, ν_as_CH_3_, and ν_as’_CH_3_ modes involved).

A separate discussion should be developed around the overtones of νC=O mode, which have attracted attention in literature, with e.g., the C=O stretching second overtone identified as a relatively well-resolved peak observed in NIR spectra of acetone and 2-hexanone and poli-3-hydroxybutyrate [76,85]. The intensity of the second overtone of C=O stretching was found to be particularly strong in relation to the corresponding first overtone for these systems [76]. As investigated in the present study, the calculated intensity of 3νC=O transition is extremely weak. There are modes of caffeine, to which the C=O stretching internal coordinate contributes meaningfully. The respective fundamental transitions appear at 1694 and 1740 cm^−1^ calculated positions. The corresponding first overtones emerge in MIR region as well, at calculated 3409 and 3314 cm^−1^. Only the second overtones appear in the NIR region, at 5101 and 4960 cm^−1^.

Let’s evaluate the calculated intensities of the first and second C=O stretching overtones in relation to the respective fundamentals. The calculated peak positions and intensities are presented in Table 3. The first overtones of C=O stretching are over 286 and 84 times weaker than the corresponding fundamental bands. The second overtones are over 3000 times weaker than the respective fundamentals, and over 11 and 46 times weaker than the first overtones. Therefore, in the case of caffeine, most likely because of the symmetry of the molecule, the second overtones of C=O stretching do not appear as meaningful peaks that could be observed in the NIR spectrum (Figure S9 in Supplementary Materials).

### 2.3. Linking the Interpreted NIR Absorption Regions of Caffeine with Wavenumber Ranges Found to Be Meaningful in Quantitative Analytical Applications

Applied NIR spectroscopic studies related to coffee analysis and quality control, performed in combination with multivariate classification and/or regression analysis for either in pure or blend samples, have put considerable attention on the absorption features of caffeine. These studies noticed the relevance for the analytical performance of the characteristic NIR bands of caffeine confronted with the other chemical constituents abundant in the sample, e.g., lipids, carbohydrates theobromine, theophylline, chlorogenic acid, or trigonelline [86]. The importance of selecting the informational spectral ranges, representing the variables in multivariate models, for the performance of such analysis was reported by Pizarro’s group [84]. Barbin et al. [51] assembled a comprehensive table of the NIR absorption regions of caffeine altogether with the other relevant chemical constituents present in coffee. The importance of a proper variable selection during the analytical procedure of model calibration, which would minimize the overlap between the absorption bands of pure components was noted as keen for obtaining more reliable analysis [51].

With the results of the present work, it becomes possible to interpret these wavenumber regions of caffeine as meaningful to the performance of analytical applications, and link them with the molecular structural features. A short discussion can be provided confronting the unveiled picture with that commonly presented in the literature, where the absorption regions are often divided to the “first, second and third overtones” regions [51,74].

Estabean-Diez et al. [87] provided assignments for some NIR bands of caffeine. For instance, the peak at 1710 nm (5848 cm^−1^) was identified as the first overtone CH_3_ asym. stretching, which can be confirmed by the present results. It should be more precisely described as CH_3_ asym. stretching', according to Pulay’s convention. The meaningful peak at 1340 nm (7463 cm^−1^) was also identified correctly as 2νCH + δCH. However, the interpretation provided for the other variables, e.g., at 1154 nm (8667 cm^−1^), 1914–1916 nm (5225–5219 cm^−1^), and 2142–2150 nm (4469–4651 cm^−1^), each identified as second overtones, should be reconsidered. These features are rather ternary combinations, as the respective spectral regions solely originate from those transitions (Figure 3 and Figure 4).

Zhang et al. [86] used three methods, within which the variables meaningful for the analysis of caffeine content have been determined. The unveiled variables concentrated in four wavenumber regions, 4019–4196, 4412–5056, 5577–6106, and 6784–7706 cm^−1^. It was anticipated that the characteristic absorption peaks of caffeine appear there relatively less obstructed from the peaks of other constituents. However, no interpretation of these spectral regions was given. Here, we can associate these meaningful variables primarily with binary and ternary combinations of various modes, primarily νCH and δCH (4019–4196 cm^−1^ and 4412–5056 cm^−1^), first overtones of δring, δCH, and ν_as_CH_3_ together with binary and ternary combinations of primarily νCH and δring (5577–6106 cm^−1^), and ternary combinations mostly involving νCH, δCH, and δring (6784–7706 cm^−1^).

Santos et al. [88] concluded from their analysis of the PLS regression coefficients for four calibrated models that the 5000–4000 cm^−1^ region is the most important for caffeine quantification. That spectral region was predominantly correctly associated with νCH and νCC combination bands. However, this interpretation should be extended to numerous other contributions from binary and ternary combination bands present in this strongly convoluted fragment of the spectrum.

Barabin et al. [51] has summarized the meaningful wavenumber regions for caffeine reported in the literature, as well as provided more generalized spectra-structure correlations. Interestingly, the absorption regions identified as meaningful for caffeine analysis by NIR spectroscopy include the weaker bands of this compound, e.g., in ca. the 5300–5000 cm^−1^ region elucidated as ternary combinations in this work. Additionally, the wavenumber regions meaningful for caffeine analysis that do not contain peaks of this compound may nevertheless carry the information correlated with caffeine content in the sample. The corresponding spectral signal may arise from the surrounding matrix molecules influenced by the interaction with caffeine.

## 3. Materials and Methods

### 3.1. Experimental

Caffeine standard (powder; ≥95%) was ordered from Extrasynthese (Genay, France). Carbon tetrachloride (CCl_4_, anhydrous, ≥99.5%) was purchased from Sigma Aldrich (St. Louis, MO, USA). Carbon tetrachloride was additionally distilled and stored over molecular sieves (5 Å, Sigma-Aldrich).

NIR spectra were measured on a Büchi NIRFlex N-500 Fourier Transform (FT) spectrometer based on a polarization interferometer. The instrument was equipped with the accessory for measuring liquid samples in transmission mode. The samples were placed in a quartz cuvette (Hellma QX) with the optical path of 10 mm. Spectra were obtained in the wavenumber range from 10,000 to 4000 cm^−1^, with a spectral resolution of 8 cm^−1^ further interpolated by the software controlling the spectrometer (Büchi NIR Ware 1.4.30010) to 4 cm^−1^, resulting in 1501 data points per spectrum. A total of 64 mean scans were collected for each spectrum. For further analysis, the spectrum of the cell filled with the solvent was subtracted from the spectra of the solution.

The spectrometer can stabilize the sample temperature during the measurements, in the range from room temperature to 338 K (65 °C). It was found that the solubility curve of caffeine in carbon tetrachloride changes noticeably with the temperature. Under normal conditions the solubility is low, resulting in a weak intensity of the measured NIR bands. To obtain better quality of the spectra, measurements of saturated solution were performed at increased temperatures up to the limit of the spectrometer of 333.15 K (60 °C), which remains at safe distance from the boiling point of carbon tetrachloride [349.85 K (76.7 °C)] as well as the temperature of decomposition of caffeine [558.15 K (285 °C)] [89]. The solubility of caffeine at that temperature increased significantly, yielding a much better resolved spectrum that enabled reliable analysis of weak bands such as those originating from the second overtones and ternary combinations.

### 3.2. Computational Details

**Figure 5:**
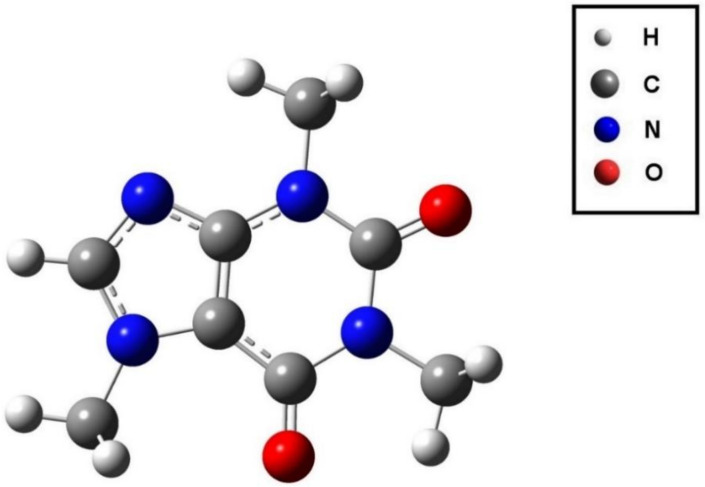
Molecular structure of caffeine optimized at B3LYP-D3/SNSD level of theory.

For the purpose of theoretical simulation of the NIR vibrational bands, anharmonic calculations by means of Generalized Vibrational Second-Order Perturbation Theory (GVPT2) were performed [90,91]. Within this approach, a full treatment of vibrational resonances is performed, with the tightly coupled states being treated with variational procedure [91]. The anharmonic vibrational analysis yielded the transitions up to three quanta; i.e., the first (2*ω*) and second overtones (3*ω*), as well as binary (*ω*_a_ + *ω*_b_) and ternary combinations of two kinds (*ω*_a_ + *ω*_b_ + *ω*_c_ and 2*ω*_a_ + *ω*_b_) were obtained. The vibrational calculations were preceded by the mandatory geometry optimization (i.e., energy minimization) step (Figure 5). The computations were carried out at Density Functional Theory (DFT) level of electronic theory, with Becke’s three-parameter exchange (B3) + Lee-Yang-Parr (LYP) density functional (i.e., B3LYP), and were additionally refined by applying Grimme’s third version of empirical correction for dispersion (GD3) [92]. A “spectroscopic” basis set of double-z quality (SNSD) developed by Barone’s group was used [93]. This combination of density functional and basis sets yielded very accurate yet reasonably time-extensive calculations of NIR bands in previous studies [31]. All quantum mechanical calculations were performed with Gaussian 16 Rev. B01 software [94]. The modeling of the spectral lineshape was carried out through parameterized band broadening. Lorentz-Gauss (Cauchy-Gauss) product function was used as the bandshape model [34,95]. The band identification and association to the vibrational modes was performed in accordance with Pulay et al. [96].

## 4. Conclusions

NIR spectrum of caffeine was successfully reproduced by anharmonic quantum chemical calculations. The calculated first and second overtones as well as binary and ternary combinations unveiled a strongly convoluted spectral lineshape. High contribution to the NIR spectrum of combination bands, including ternary combinations, was noticed in the entire studied spectral region, and between 7000–4000 cm^−1^ in particular. Noteworthy, the region between 5000–4000 cm^−1^ is dominated by the combination bands, but in the case of caffeine, significant input to the NIR spectral intensity in that region can be observed from both binary and ternary combination bands. The contributions from the overtones to the NIR spectrum of caffeine ranges widely depending on a particular fragment of the spectrum. The first overtones are mostly manifested in the 6500–5500 cm^−1^ region, while the second overtones dominate the upper wavenumber (i.e., shorter wavelength) region, between ca. 9200–8400 cm^−1^. The present study demonstrates theoretically that NIR spectra are highly region specific with respect to the first and second overtones as well as binary and ternary combination bands. Following a detailed analysis of vibrational transition and band assignments, the interpterion of the wavenumber regions meaningful for caffeine analysis by NIR spectroscopy was performed and discussed with the previous findings reported in literature, which were based on conventional methods of spectra analysis.

## Figures and Tables

**Table 1:** Vibrational contributions to NIR spectrum (in % of the integral intensity) of caffeine in selected wavenumber regions based on the calculated spectrum.

Region [cm^−1^]	First Overt.	Second Overt.	Binary Comb.	Ternary Comb.
10,000–4000	11.57	1.28	36.91	50.24
10,000–7000	0.02	26.78	0.00	73.20
7000–4000	11.85	0.64	37.83	49.67
5000–4000	0.01	0.88	48.10	51.01
6500–5500	40.52	0.00	14.77	44.70
7700–7000	0.05	0.00	0.01	99.95
9200–8400	0.00	42.27	0.00	57.73

**Table 2:** Summarized the assignments for the major NIR peaks of caffeine based on GVPT2//B3LYP-GD3BJ/SNSD calculations.

Position [cm^−1^]		Assignments
Exp.	Calc.	
4092	4062	δ_rock_CH_3_ + ν_s_CH_3_; (δ_ip_ring, δ_ip_CH, δ_rock_CH_3_) + ν_as_CH_3_
4112	4096	δ_rock_CH_3_ + ν_as_CH_3_; CN_tors_ + ν_as_CH_3_
4183	4181	(δ_ip_CH, δ_ip_ring) + ν_s_CH_3_; CN_tors_ + δ_ip_ring + νCH
4236	4227	(δ_ip_ring, δ_ip_CH) + ν_as_CH_3_; δ_rock_CH_3_ + νCH; δ_ip_ring + ν_s_CH_3_
4308	4282	(δ_ip_CH, δ_ip_ring) + ν_s_CH_3_; (δ_ip_CH, δ_ip_ring) + νCH
4428	4376	ν_as’_CH_3_ + δ_s_CH_3_; δ_as_CH_3_ + ν_as’_CH_3_; δ_s_CH_3_ + ν_s_CH_3_
4444	4434	δ_s_CH_3_ + ν_as_CH_3_; ν_as’_CH_3_ + δ_ip_ring + δ_rock_CH_3_; (δ_ip_ring, δ_ip_CH) + νCH
5760	5761	CN_tors_ + ν_as’_CH_3_ + ν_s_CH_3_
5800	5815	ν_as’_CH_3_ + ν_s_CH_3_
5840	5881	2ν_as_CH_3_
5956	5973	ν_as’_CH_3_ + ν_as_CH_3_; CN_tors_ + ν_as’_CH_3_ + ν_s_CH_3_
6008	5991	2ν_as’_CH_3_
6120	6111	2νCH
7140	7211	ν_as’_CH_3_ + δ_as_CH_3_ + ν_s_CH_3_
7236	7239	ν_as’_CH_3_ + δ_as_CH_3_ + ν_s_CH_3_
7300	7282	δ_ip_CH, δ_ip_ring + 2νCH
7344	7321	2ν_as’_CH_3_ + δ_as_CH_3_
7372	7355	δ_as_CH_3_ + 3ν_as_CH_3_; δ_s_CH_3_ + 3ν_as_CH_3_
8532	8610	2ν_as’_CH_3_ + v_s_CH_3_; ν_as’_CH_3_ + 2ν_s_CH_3_
8716	8752	ν_as’_CH_3_ + ν_s_CH_3_ + ν_as_CH_3_; ν_as’_CH_3_ + ν_s_CH3 + νCO; 3ν_as’_CH_3_; ν_s_CH_3_ + 2ν_as_CH_3_
8812	8830	3ν_as_CH_3_
8980	8984	3νCH

Where: υ—stretching, δ—bending, tors—torsion, rock—rocking, oop—out-of-plane, ip—in-plane, s—symmetric, as—antisymmetric.

**Table 3:** Calculated (GVPT2//B3LYP-GD3BJ/SNSD) positions and intensities of the bands corresponding to two modes of caffeine, in which the C=O stretching coordinate is meaningful.

	ν_1_C = O		2ν_1_C = O		3ν_1_C = O	
	Position [cm^−1^]	Intensity [km mol^−1^]	Position [cm^−1^]	Intensity [km mol^−1^]	Position [cm^−1^]	Intensity [km mol^−1^]
relative intensity	1740	206.4	3409.3	0.72	5100.9	0.064
		1		0.0035		0.00031
				1		0.089
	**ν_2_C = O**		**2ν_2_C = O**		**3ν_2_C = O**	
relative intensity	1694	83.4	3313.6	0.99	4959.9	0.02127
		1		0.011871		0.00026
				1		0.021

## Data Availability

Not applicable.

## Supplementary Materials

The following are available online, Figure S1: Experimental and calculated NIR spectra of caffeine in region 7000–4000 cm^−1^. In addition to the theoretical lineshape, individual simulated bands are presented as well; Figure S2: Experimental and calculated NIR spectra of caffeine in region 5000–4000 cm^−1^. Theoretical lineshapes representing the summed bands of different origin are presented; Figure S3: Experimental and calculated NIR spectra of caffeine in region 4800–4000 cm^−1^. In addition to the theoretical lineshape, individual simulated bands are presented as well; Figure S4: Experimental and calculated NIR spectra of caffeine in region 5700–4800 cm^−1^. Theoretical lineshapes representing the summed bands of different origin are presented; Figure S5: Experimental and calculated NIR spectra of caffeine in region 7000–5000 cm^−1^. Theoretical lineshapes representing the summed bands of different origin are presented; Figure S6: Experimental and calculated NIR spectra of caffeine in region 7000–5000 cm^−1^. In addition to the theoretical lineshape, individual simulated bands are presented as well; Figure S7: Experimental and calculated NIR spectra of caffeine in region 9500–8000 cm^−1^. Theoretical lineshapes representing the summed bands of different origin are presented; Figure S8: Experimental and calculated NIR spectra of caffeine in region 9500–8000cm^−1^. In addition to the theoretical lineshape, individual simulated bands are presented as well; Figure S9: The calculated contributions to NIR spectrum of caffeine of C=O stretching transitions.

## Sample Availability

Samples of the compounds in this article are not available from the authors.
