# Supplementary material for: Anharmonic DFT Study of Near-Infrared Spectra of Caffeine: Vibrational Analysis of the Second Overtones and Ternary Combinations
Source: Molecules. 2021 Aug 27;26(17):5212. doi: 10.3390/molecules26175212 (PMC8433751; doi:10.3390/molecules26175212)
Supplement: Supplementary file 1 [file molecules-26-05212-s001.zip › molecules-1338179-supplementary.pdf]

Anharmonic DFT Study of Near-infrared Spectra of Caffeine. Vibrational Analysis of the Second Overtones and Ternary Combinations

SUPPLEMENTARY MATERIALS

Justyna Grabska,[1*] Krzysztof B. Beć,[1] Yukihiro Ozaki,[2,3] Christian W. Huck[1]

[1] Institute of Analytical Chemistry and Radiochemistry, Leopold-Franzens University, Innrain 80/82, CCB-Center for Chemistry and Biomedicine, 6020 Innsbruck, Austria

[2] School of Biological and Environmental Sciences, Kwansei Gakuin University, Sanda, Hyogo 669-1337, Japan

[3]Toyota Physical and Chemical Research Institute, Yokomichi, Nagakute, Aichi 480-1192, Japan

*Corresponding author: Justyna Grabska (Justyna.Grabska@uibk.ac.at)

Institute of Analytical Chemistry and Radiochemistry, Leopold-Franzens University, Innrain 80/82, CCB-Center for Chemistry and Biomedicine, 6020 Innsbruck, Austria

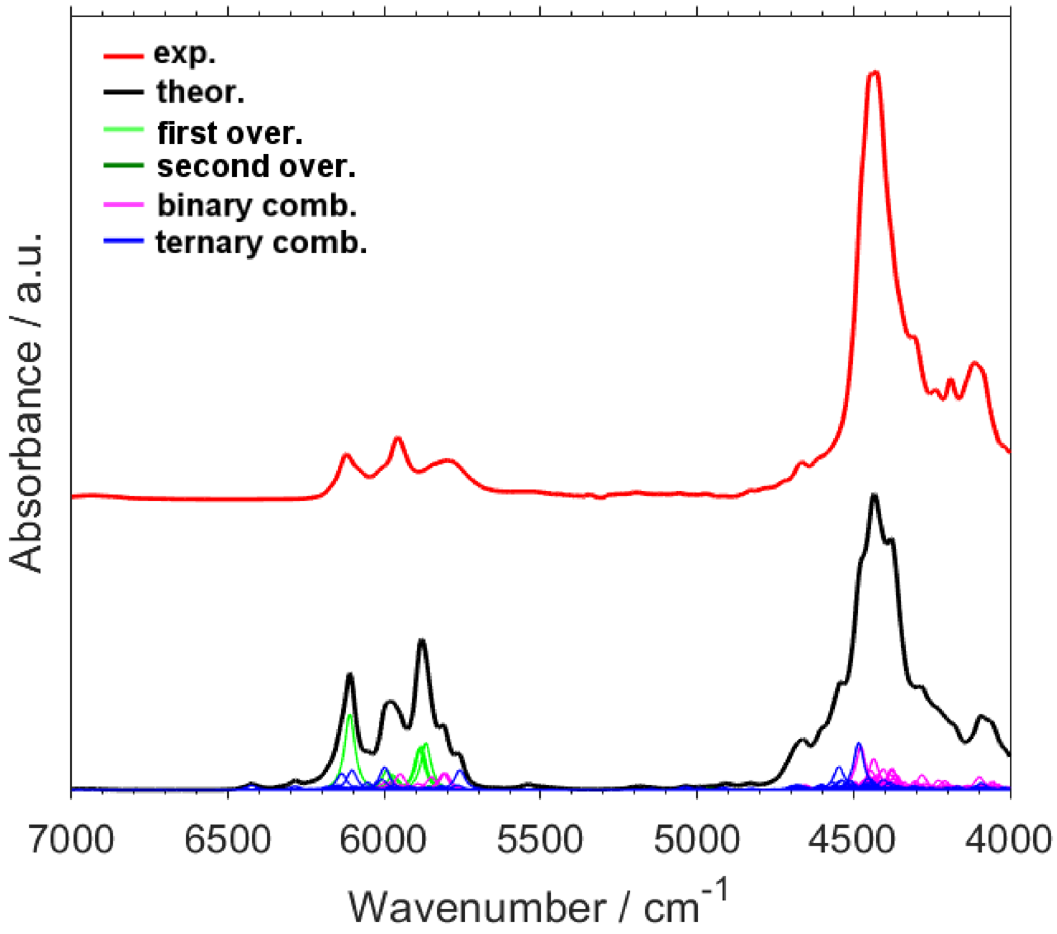

Figure S1. **Experimental and calculated NIR spectra of caffeine in region 7000-4000 cm^{-1}. In addition to the theoretical lineshape, individual simulated bands are presented as well.**

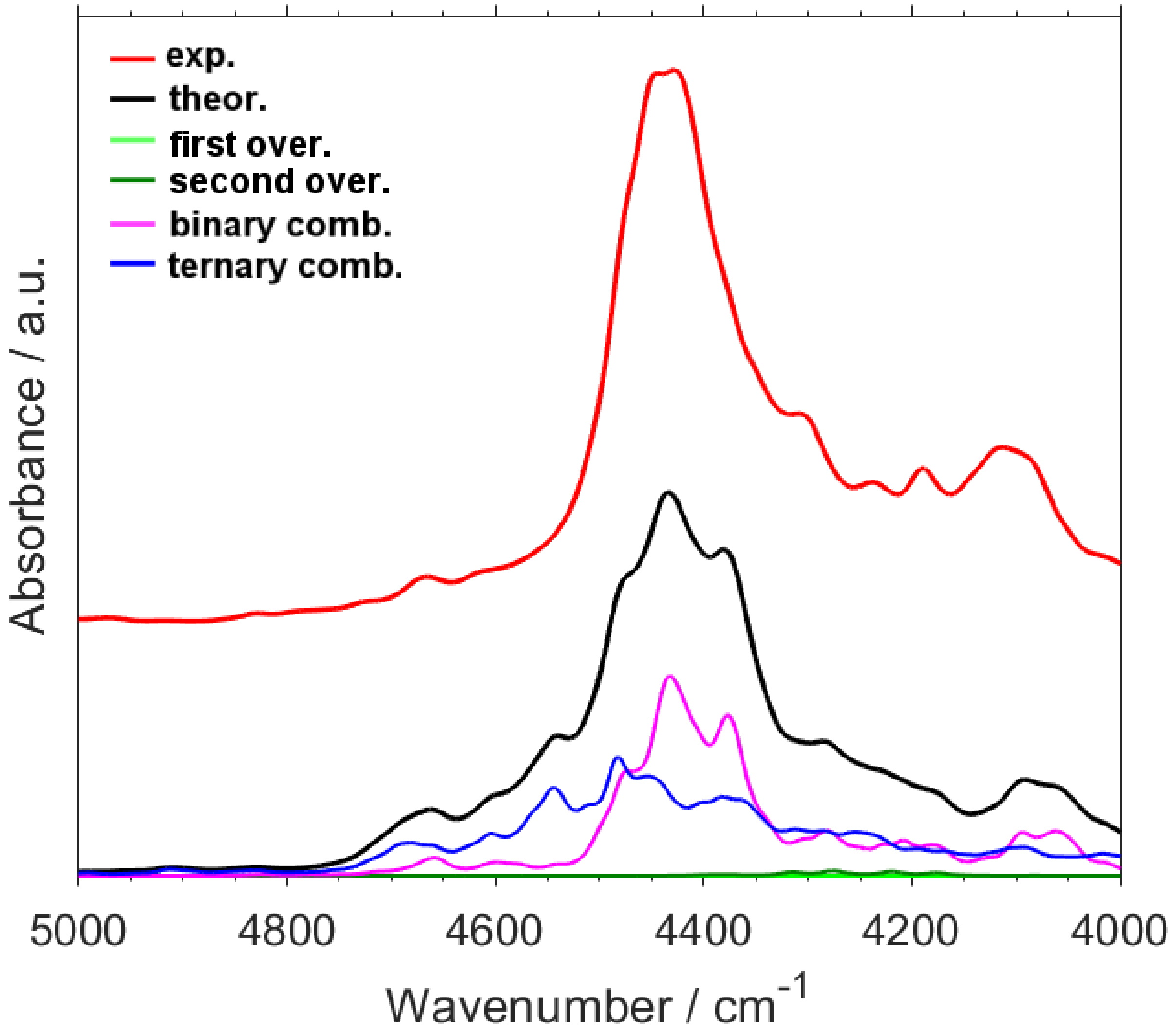

Figure S2. **Experimental and calculated NIR spectra of caffeine in region 5000-4000 cm^{-1}. Theoretical lineshapes representing the summed bands of different origin are presented.**

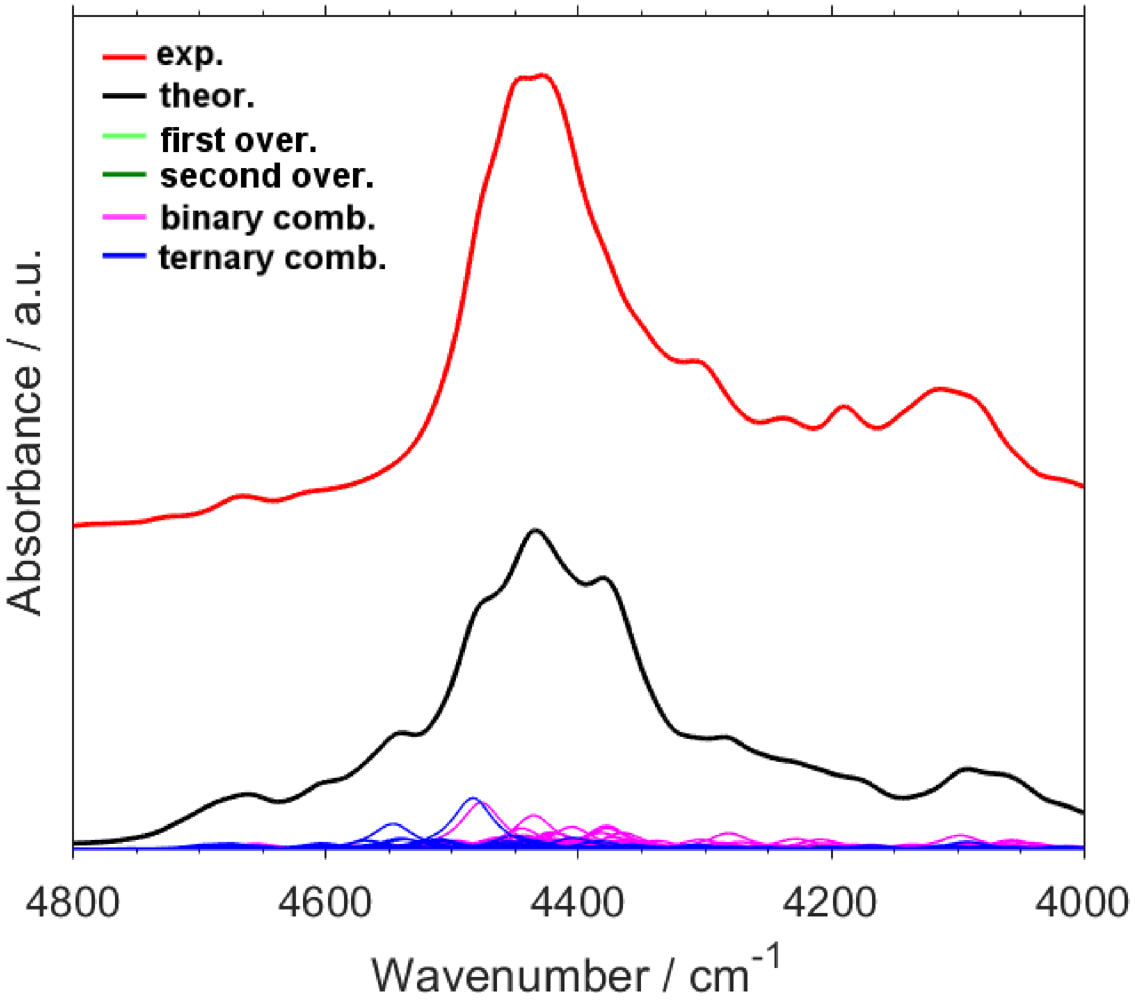

Figure S3. Experimental and calculated NIR spectra of caffeine in region 4800-4000 cm^{-1}. In addition to the theoretical lineshape, individual simulated bands are presented as well.

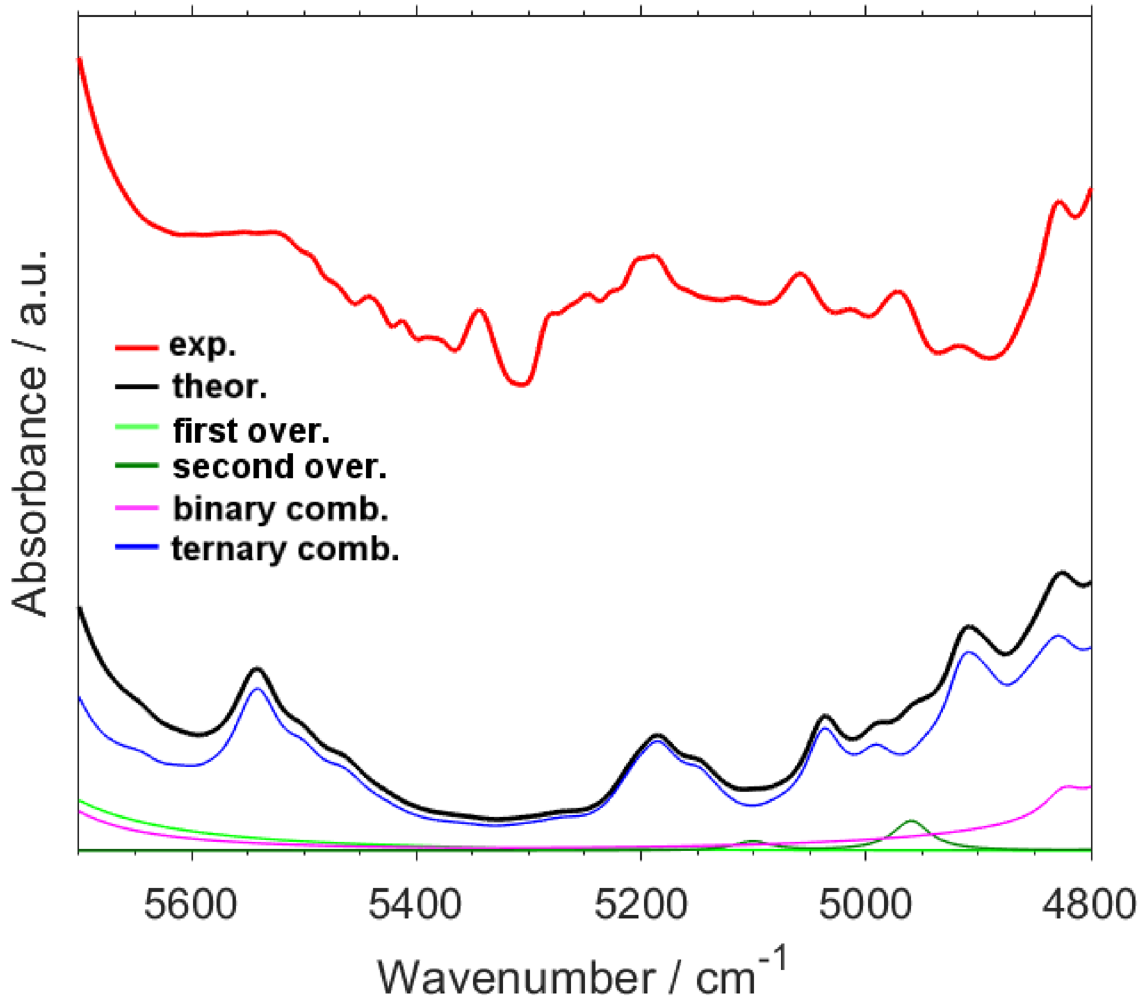

Figure S4. **Experimental and calculated NIR spectra of caffeine in region 5700-4800 cm^{-1}. Theoretical lineshapes representing the summed bands of different origin are presented.**

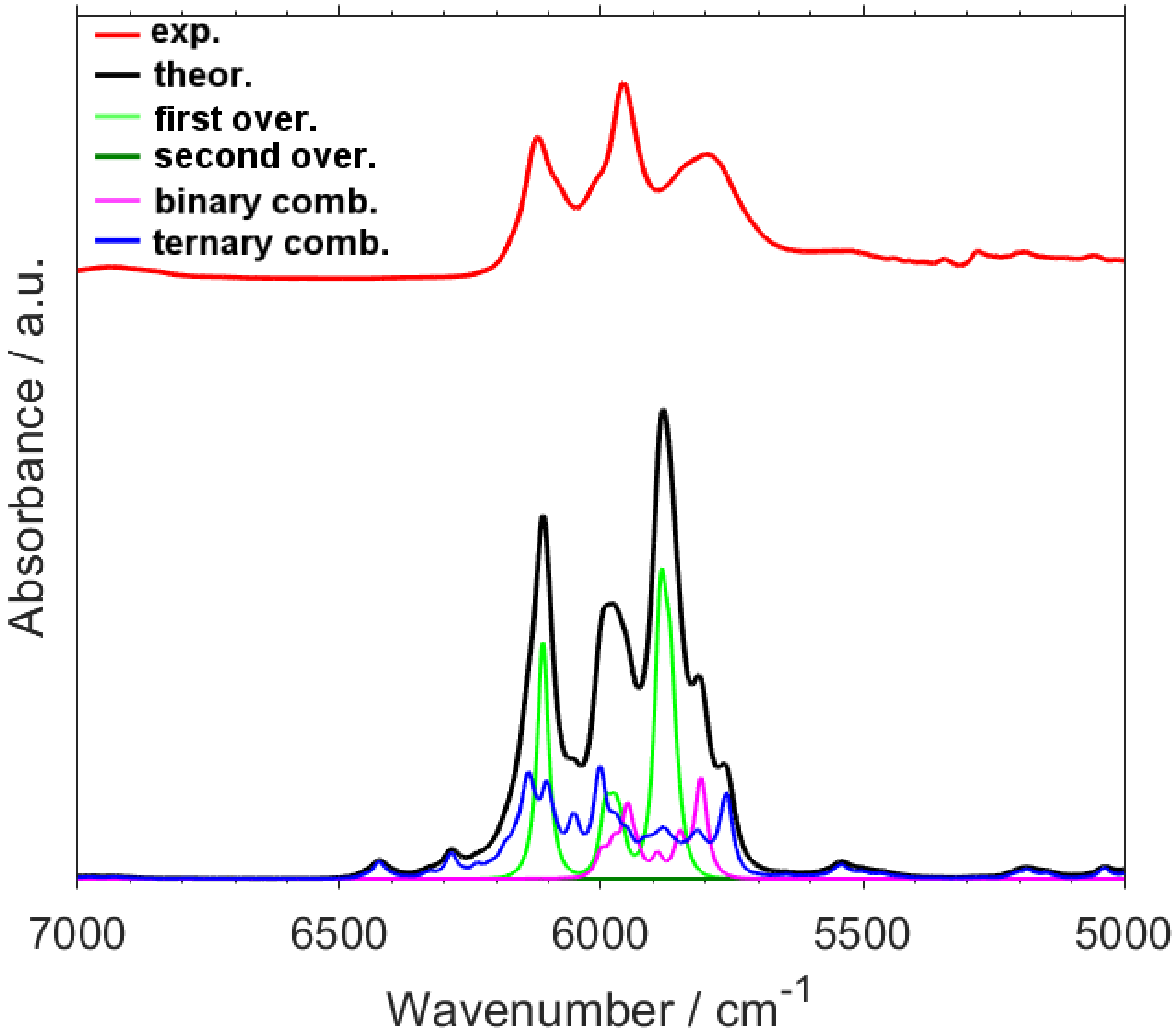

Figure S5. Experimental and calculated NIR spectra of caffeine in region 7000-5000 cm^{-1}. Theoretical lineshapes representing the summed bands of different origin are presented.

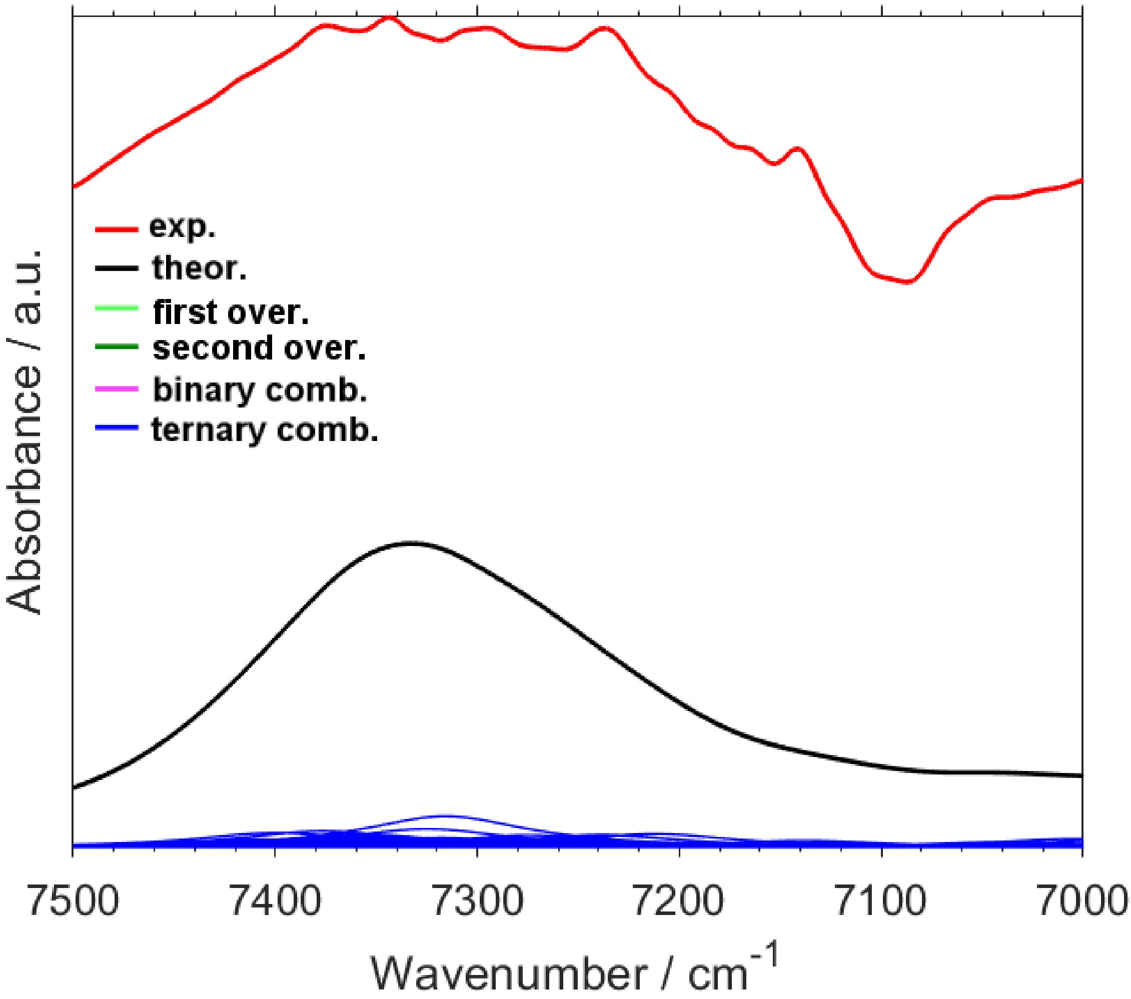

Figure S6. Experimental and calculated NIR spectra of caffeine in region 7000-5000 cm^{-1}. In addition to the theoretical lineshape, individual simulated bands are presented as well.

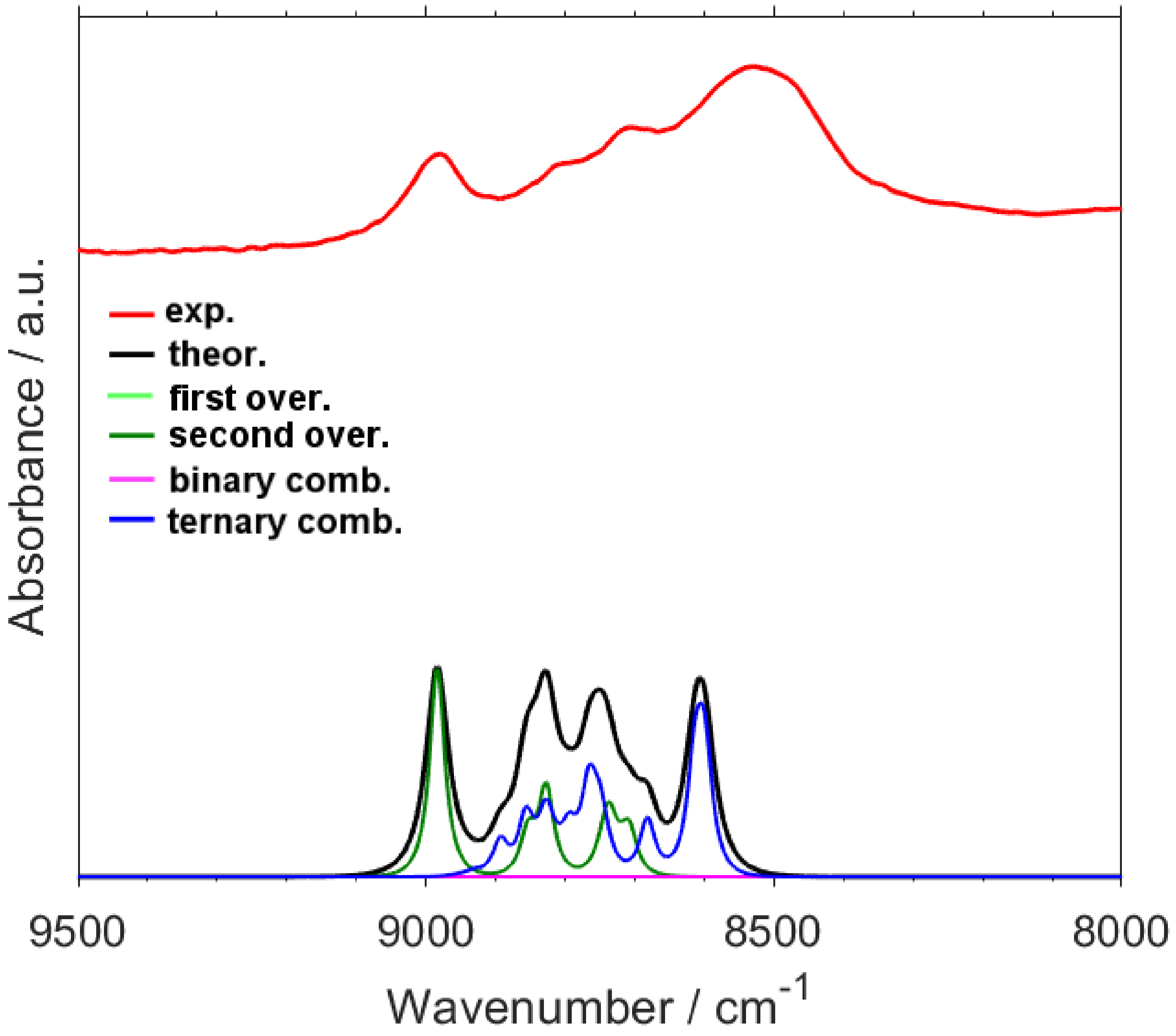

Figure S7. Experimental and calculated NIR spectra of caffeine in region 9500-8000 cm^{-1}. Theoretical lineshapes representing the summed bands of different origin are presented.

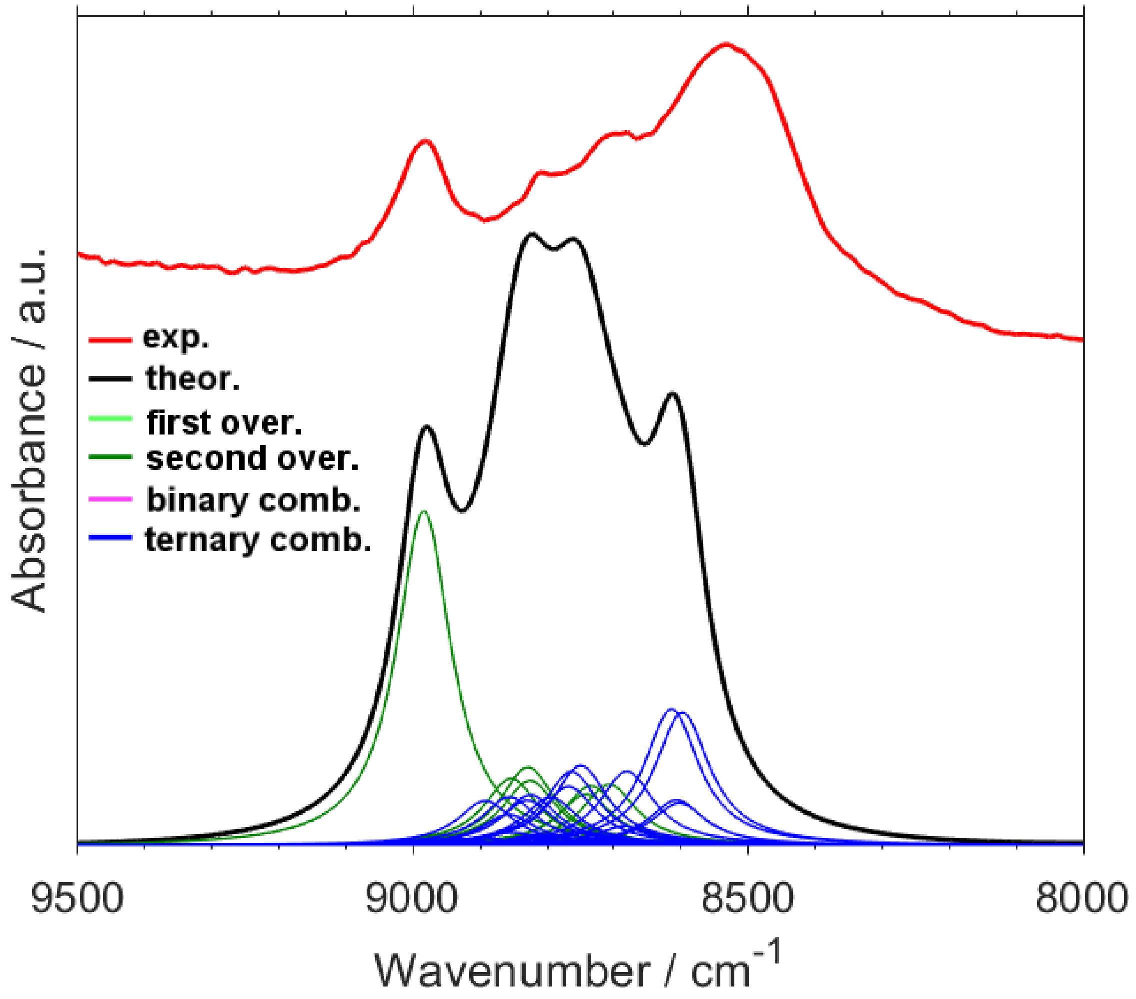

Figure S8. Experimental and calculated NIR spectra of caffeine in region 9500-8000cm^{-1}. In addition to the theoretical lineshape, individual simulated bands are presented as well.

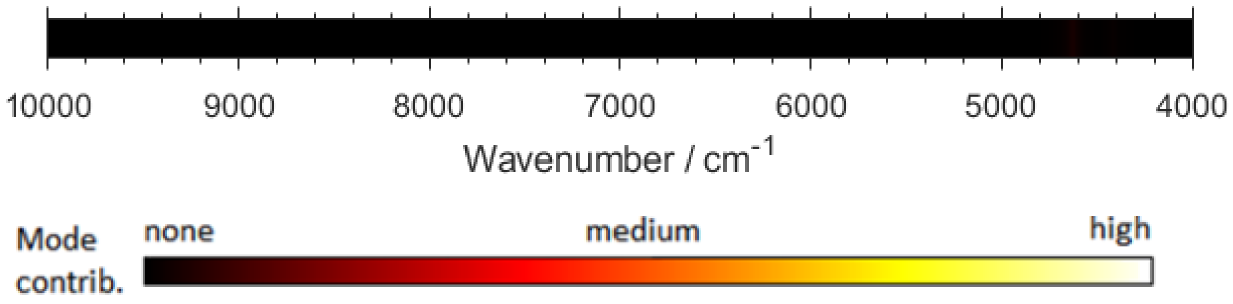

Figure S9. The calculated contributions to NIR spectrum of caffeine of C=O stretching transitions.

Funding

This work was supported by the Austrian Science Fund (FWF): P32004-N28.

Acknowledgement

Calculations have been carried out in Wrocław Centre for Networking and Supercomputing (http://www.wcss.pl), under grant no. 375.
